# Porcine Epidemic Diarrhea Virus (PEDV) ORF3 Interactome Reveals Inhibition of Virus Replication by Cellular VPS36 Protein

**DOI:** 10.3390/v11040382

**Published:** 2019-04-24

**Authors:** Challika Kaewborisuth, Yodying Yingchutrakul, Sittiruk Roytrakul, Anan Jongkaewwattana

**Affiliations:** 1 Virology and Cell Technology Laboratory, National Center for Genetic Engineering and Biotechnology (BIOTEC), National Science and Technology Development Agency (NSTDA), Pathumthani 12120, Thailand; challika.kae@biotec.or.th; 2 Proteomics Research Laboratory, National Center for Genetic Engineering and Biotechnology (BIOTEC), National Science and Technology Development Agency (NSTDA), Pathumthani 12120, Thailand; yodying.yin@biotec.or.th (Y.Y.); sittiruk@biotec.or.th (S.R.)

**Keywords:** Porcine epidemic diarrhea virus, PEDV, ORF3 accessory protein, interactome, VPS36, virus replication

## Abstract

The accessory protein ORF3 of porcine epidemic diarrhea virus (PEDV) has been proposed to play a key role in virus replication. However, our understanding of its function regarding virus and host interaction is still limited. In this study, we employed immunoprecipitation and mass spectrometry to screen for cellular interacting partners of ORF3. Gene ontology analysis of the host interactome highlighted the involvement of ORF3 in endosomal and immune signaling pathways. Among the identified ORF3-interacting proteins, the vacuolar protein-sorting-associated protein 36 (VPS36) was assessed for its role in PEDV replication. VPS36 was found to interact with ORF3 regardless of its GLUE domain. As a result of VPS36–ORF3 interaction, PEDV replication was substantially suppressed in cells overexpressing VPS36. Interestingly, the ORF3 protein expression was diminished in VPS36-overexpressing cells, an effect that could not be restored by treatment of lysosomal inhibitors. In addition, disruption of endogenously-expressed VPS36 by siRNA could partially augment PEDV replication. Taken together, our study provides mechanistic insights into the contribution of ORF3 in PEDV replication.

## 1. Introduction

Porcine epidemic diarrhea virus (PEDV) is one of the major enteric pathogens currently threatening the swine population worldwide. Infection of PEDV, especially in neonatal piglets, leads to severe watery diarrhea, dehydration, and death, resulting in tremendous economic losses [1,2]. PEDV belongs to the *Alphacoronavirus* genus in the Coronaviridae family. Its genome is a positive-sense single-stranded RNA encoding for viral replicase polyprotein (pp) la and pp1ab, spike (S), accessory protein (ORF3), envelope (E), membrane (M), and the nucleocapsid (N) proteins [3]. Like other coronaviruses (CoVs), PEDV initiates its replication cycle when the S protein binds to the cellular receptor. Shortly after receptor binding, the S protein undergoes conformational changes that facilitate the fusion between viral envelope and cell or endosomal membrane. Subsequently, PEDV releases genomic RNA into the cytosol of the host cell to initiate the translation of replicase polyproteins pp1a and 1ab followed by cleavage into small products by viral proteases. Concurrently, subgenomic mRNAs are generated by discontinuous transcription and translated to structural proteins. Nascent genomic RNA and structural proteins are assembled into new virions in the ER-Golgi intermediate compartment (ERGIC) and subsequently released out of the host cell via exocytosis [4].

While the biology of PEDV structural proteins can be inferred from those of well-characterized relatives, such as transmissible gastroenteritis virus (TGEV), severe acute respiratory syndrome coronavirus (SARS-CoV), or Middle East respiratory syndrome coronavirus (MERS-CoV), knowledge on the role of the ORF3 accessory protein in PEDV replication is limited. Although ORF3 has recently been proposed to play a role in regulating PEDV virus replication and pathogenesis [5,6,7], its interaction with the host cell’s proteins has not been clearly addressed. ORF3 has been shown to induce formation of double-membrane vesicles and prolong S phase of a cell cycle in transfected cells [8]. Moreover, our previous study demonstrated that ORF3 is localized in diverse cellular compartments, including cell surface, endoplasmic reticulum (ER), and Golgi apparatus (Golgi), and cooperated with the S protein to support virus replication in cell culture [9]. These findings point to the possibility that ORF3 engages in a variety of cellular pathways, some of which might be important for PEDV replication. Immunoprecipitation coupled with mass spectrometry is a combination method commonly used to identify novel viral-host protein–protein interaction [10,11,12]. Thus, identification of the ORF3 interactome would enable more insights into PEDV–host interplays.

In this study, we employed immunoprecipitation and liquid chromatography–tandem mass spectrometry (LC-MS/MS) to discover the interactome of ORF3. Our study provides important insights into PEDV–host protein interaction, especially into the role of ORF3 in the endosomal and immune signaling pathways. Our data presented here also underscore the ubiquitination of ORF3 and the role of the cellular VPS36 protein in impeding PEDV growth.

## 2. Materials and Methods

### 2.1. Cell Line and Plasmid Construction

Human embryonic kidney (HEK) 293T ((ATCC CRL-3216) and African green monkey (VeroE6) cells (ATCC CRL-1586) stably expressing porcine aminopeptidase N (VeroE6-APN) [13] were grown and maintained in Opti-MEM^TM^ (Gibco^TM^, Thermo Scientific, Waltham, MA, USA) supplemented with 10% heat-inactivated fetal bovine serum (FBS). Cells were cultured in humidified air containing 5% CO_2_ at 37 °C.

pCAGGS expressing the full-length PEDV ORF3 protein with myc-tag at the C-terminus (ORF3myc) was constructed as previously described [9]. Cellular ORF3-interacting proteins, vacuolar protein-sorting-associated protein 36 (VPS36: NM_016075.3), and inhibitor of nuclear factor kappa-B kinase subunit beta (IKBKB: NM_001556.2) with flag-tag at the C-terminus were synthesized and cloned into pCAGGS plasmids. All plasmids were subject to direct nucleotide sequencing (First Base, Selangor, Malaysia) and western blot analysis for protein expression. 

### 2.2. Co-Immunoprecipitation

HEK293T cells in a 100 mm^2^ tissue culture plate at 70% cell confluence were transfected with pCAGGS empty or pCAGGS expressing ORF3myc using Fugene HD transfection reagent (Promega, Madison, WI, USA) following the manufacturer’s instructions. At 24 h post transfection (hpt), cells were washed with cold phosphate-buffer saline (PBS) and re-suspended in pre-cooled IP lysis buffer (Pierce^TM^, Thermo Scientific, Waltham, MA, USA) supplemented with protease inhibitor cocktail (Thermo Scientific). Cell lysates were cleared by centrifugation at 14,000× *g* for 5 min at 4 °C. Cleared lysates were then incubated with anti-myc agarose (Pierce^TM^, Thermo scientific) with gentle rocking overnight at 4 °C. Immunoprecipitates were washed with TBST buffer (25 mM Tris-HCl, 0.15 M NaCl, 0.05% tween 20, pH 7.2) and eluted in SDS sample loading buffer. The samples were subjected to SDS-PAGE. Gels were subsequently stained by silver staining for mass spectrometry (Pierce^TM^, Thermo scientific). 

HEK293T cells were co-transfected with pCAGGS_ORF3myc and pCAGGS_VPS36flag or VPS36 fragments (F1; amino acids 1–139 and F2; amino acids 140–387) (Figure 4a). At 24 hpt, the transfected cells were prepared for immunoprecipitation as described above. Immunoprecipitates were eluted in sample buffer followed by SDS-PAGE and western blot.

To determine ubiquitination of ORF3, precipitation of expressed HA-Ub proteins using affinity purification was performed as described previously [14] with some modifications. Briefly, HEK293T cells were transfected with plasmids expressing ubiquitin with HA-tag at N-terminus (HA-Ub) and ORF3myc or empty plasmid. Cells were harvested for immunoprecipitation and western blot analysis as described above. Rabbit anti-HA tag (Abcam Cambridge, MA, USA) and anti-myc antibodies (Abcam) were used to detect ubiquitin and ORF3.

### 2.3. In-gel Digestion and Mass Spectrometry

The silver staining gel was divided into 10 parts. Each excised band was cut into small cubes and digested as previously described [15]. Briefly, gels were destained with 3% sodium peroxide (NaO_2_) and subjected to dehydration in 100% acetonitrile (ACN). Samples were then incubated with 10 mM dithiothreitol (DTT) in 10 mM NH_4_HCO_3_ at 56 °C for sulfhydryl bond reduction. For the alkylation step, 100 mM iodoacetamide in 10 mM NH_4_HCO_3_ was added into the samples and incubated in the dark at room temperature (RT) for 45 min. Samples were then dehydrated with 100% ACN by shaking at RT for 5 min. Sequencing-grade trypsin (Promega, Madison, WI, USA) was added to the gels and incubated at 37 °C overnight for in-gel digestion. Peptide products were extracted from the samples by adding 50% ACN in 0.1% formic acids, incubated at RT for 10 min, and dried at 45 °C for 4 h. Tryptic peptides were protonated with 0.1 % formic acid before operating the LC-MS/MS.

The tryptic peptide samples were prepared for injection into an Ultimate3000 Nano/Capillary LC System (Thermo Scientific) coupled to a Hybrid quadrupole Q-Tof impact II™ (Bruker Daltonics, Billerica, MA, USA) equipped with a Nano-captive spray ion source. Briefly, peptides were enriched on a µ-Precolumn 300 µm id × 5 mm C18 Pepmap 100, 5 µm, 100 A (Thermo Scientific), separated on a 75 μm id × 15 cm and packed with Acclaim PepMap RSLC C18, 2 μm, 100Å, nanoViper (Thermo Scientific). Solvent A and B containing 0.1% formic acid in water and 0.1 % formic acid in 80% acetonitrile, respectively, were supplied on the analytical column. A gradient of 5%–55% solvent B was used to elute the peptides at a constant flow rate of 0.30  μL/min for 30 min. Electrospray ionization was carried out at 1.6 kV using the CaptiveSpray. Mass spectra (MS) and MS/MS spectra were obtained in the positive-ion mode over the range (m/z) 150–2200 (Compass 1.9 software, Bruker Daltonics). The mass spectra were acquired using Bruker compass data analysis 4.4 software (Bruker Daltonics) and converted into mzXML format with CompassXport 3.0 software (Bruker Daltonics). DeCyder MS differential analysis 2.0 software (DeCyderMS, GE Healthcare, Cincinnati, OH, USA) was used to quantitate proteins. Protein identifications were assigned using MASCOT 2.3 software (Matrix Science, London, UK) with Bacteria database (NCBInr databank). To identify the ORF3 interactome, the data obtained from the ORF3 sample were subtracted with those of the mock control.

### 2.4. Western Blot Assay

Cells were collected and re-suspended in the Pierce^TM^ mammalian cell lysis buffer (Thermo scientific). Protein samples were mixed with SDS sample loading buffer, loaded onto 10% or 12% polyacrylamide gel, and transferred to nitrocellulose membranes. Membranes were probed with rabbit anti-myc (Abcam) antibodies to detect the ORF3 and with rabbit anti-flag (Abcam) antibody to detect VPS36- and IKBKB-flag. Mouse anti-β-actin (Cell signaling) and -myc (Invitrogen) antibodies were used to detect β-actin, and myc IgG was used for internal controls. Rabbit anti-VPS36 antibodies (Invitrogen) were used to detect endogenous VPS36 protein. Goat anti-rabbit IgG-HRP (KPL, MA, USA) and anti-mouse IgG-HRP antibodies were used as secondary antibodies for chemiluminescence detection by ChemiDoc™ XRS+ imager (BioRad, Hercules, CA, USA).

### 2.5. Virus Infection

PEDV_AV12__ORF3myc virus [9] was adsorbed onto VeroE6-APN cells grown in a 6-well plate. After incubation for 1 h, cells were washed twice with PBS and maintained in OptiMEM supplemented with recombinant trypsin (2 µg/mL) (Thermo Scientific). Cell supernatants were collected at indicated time points for virus titration.

### 2.6. Confocal Microscopy

The plasmids expressing VPS36flag or IKBKBflag and ORF3myc were co-transfected into HEK293T or VeroE6-APN cells using Fugene HD transfection reagent (Promega) following the manufacturer’s instructions. At 24 hpt, cells were fixed with 4% paraformaldehyde in PBS for 20 min at 4 °C. Subsequently, cells were washed thrice with PBS and blocked with PBS containing 10% FBS, 1% BSA, and 0.2% TritonX-100 for 1 h. Cells were then incubated for 1 h with rabbit anti-flag (Abcam) and mouse anti-myc antibodies (Invitrogen, Carlsbad, CA, USA) diluted in 10% FBS at a dilution of 1:500 and 1:500, respectively. Goat anti-rabbit IgG Alexa Fluor 488 and anti-mouse IgG Alexa Fluor 647 antibodies (both from Abcam) in 10% FBS at a dilution of 1:1000 were used as secondary antibodies and further incubated for 1 h. The glass slips were mounted on slides with ProLong Gold Antifade Mountant with DAPI (Invitrogen, Carlsbad, CA, USA). The samples were analyzed by Fluoview^TM^ FV1000 confocal microscopy (Olympus, Tokyo, Japan). 

### 2.7. Virus Titration

A monolayer of VeroE6-APN cells was grown in six-well plate and inoculated with 10-fold serial dilutions of the recombinant PEDV_AV12_ viruses. At 24 hpi, infected cells were fixed with 80% cold acetone for 10 min and washed twice with PBS. Blocking solution containing 10% FBS and 1% BSA was added and incubated at room temperature for 1 h with gentle agitation. After the blocking step, cells were incubated with mouse anti-PEDV N antibodies (Medgene, Brookings, SD, USA) and goat anti-mouse IgG alkaline phosphatase antibodies (Abcam). The syncytium-forming unit was examined based on color formation after the addition of 1-Step™ NBT/BCIP Substrate Solution (Thermo Scientific).

### 2.8. Cell viability (CCK-8) Analysis

VeroE6-APN cells grown in a 96-well plate were mock-transfected or transfected with plasmid expressing VPS36 protein at varied concentrations. Empty plasmid was used as a mock control. At 24 hpt, cell viability was measured by adding CCK-8 solution (Sigma) directly to the cells following the manufacturer’s instructions. The absorbance was measured using a microplate reader at 450 nm. The absorbance data was analyzed by comparing the absorbance value of mock-transfected and VPS36-transfected cells. The results are expressed as the mean of three independent experiments ± standard error of means (SEM).

### 2.9. Lysosomal Inhibitor Treatment

VeroE6-APN cells were transfected with plasmid expressing ORF3 and VPS36 proteins for 8 h followed by chloroquine treatment at a concentration of 20 µM for 1 h. After incubation, the drug was removed and replaced with fresh media. At 48 hpt, cells were harvested and subjected to SDS-PAGE and western blot analyses.

### 2.10. VPS36 Knock-Down by Small Interfering RNA Assay

Small interfering RNA (siRNA) targeting the VPS36 gene was designed and synthesized by using Dicer-substrate RNAi (DsiRNA) technology (IDT, CA, USA) as shown in Table 2. VeroE6-APN cells grown in six-well plates were transfected with the DsiRNA at a final concentration of 40 nM by using Fugene HD transfection reagent. After 48 hpt, cells were infected with PEDV_AV12__ORF3myc at an MOI of 0.1. At indicated time points, the viruses were collected for virus quantification. 

VPS36 expression in knock-down cells was determined by quantitative RT-PCR and western blot analysis to measure mRNA and protein expression levels. Primers used to target VPS36 mRNA are presented in Table 2. Rabbit anti-VPS36 antibody (Invitrogen) was used to detect endogenous VPS36 protein.

### 2.11. Quantitative RT-PCR (RT-qPCR)

Total RNA was isolated from VeroE6-APN cells transfected with siRNA by using RNeasy kit (Qiagen) and then subjected to RT-qPCR using Luna^®^ Universal One-Step RT-qPCR Kit (NEB, USA) according to the manufacturer’s protocol. RT-qPCR was performed using the CFX96 Touch™ Real-Time PCR Detection System (BioRad). The housekeeping gene GAPDH was used as an internal control. Normalized data from each sample relative to zero were compared by the threshold cycle (∆∆CT) method [16]. All primers used are listed in Table 2.

To determine the viral RNA copy number, the supernatant containing virus collected from infected cells at indicated time points was subjected for viral RNA isolation using a Luna^®^ Universal One-Step RT-qPCR Kit (NEB) following the manufacturer’s instructions. The primers specific for PEDV M gene and quantification of the virus copy number were described previously [7].

### 2.12. Statistical Analysis

GraphPad Prism 5.0 (GraphPad Software Inc., La Jolla, CA, USA) was used for statistical analyses. The differences in mean values of virus titer between groups were analyzed by Two-way ANOVA. All results were represented as means ± standard error of means (SEM); *p* values of < 0.05 were considered statistically significant.

## 3. Results

### 3.1. Identification of Cellular Protein Partners of PEDV ORF3

**Figure 1:**
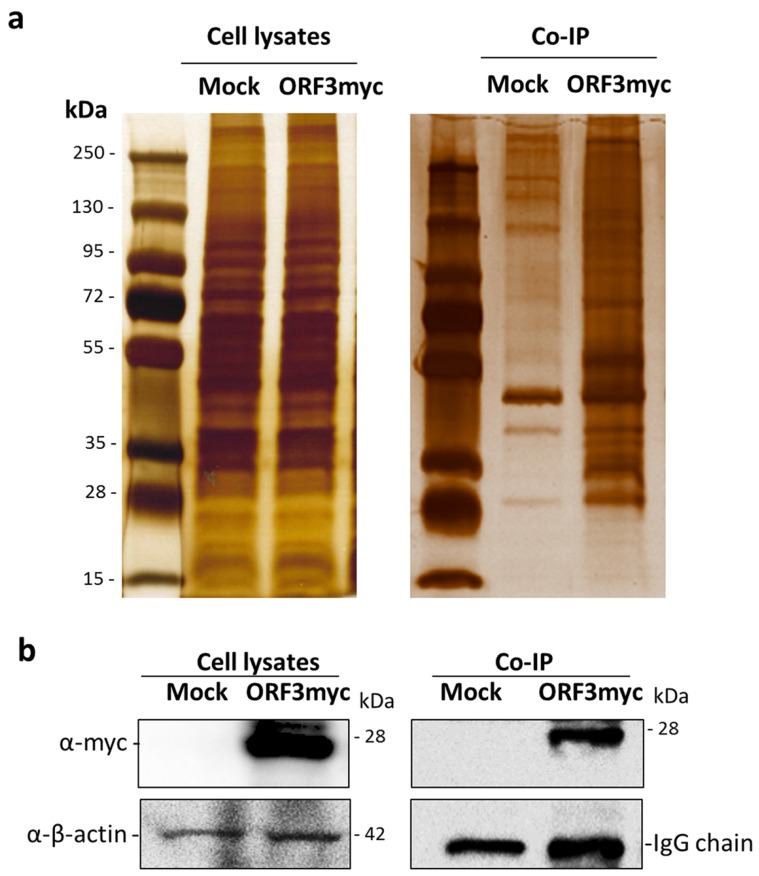
SDS-PAGE and western blot analysis of immunoprecipitated samples. (**a**) Plasmid expressing ORF3myc was transfected into HEK293T cells. Empty plasmid was used as mock transfected. At 24 hpt, cell lysates were harvested and subjected to immunoprecipitation with anti-myc affinity beads. The pull-down proteins were separated by 10% SDS-PAGE and visualized by silver staining. (**b**) The pull-down proteins were transferred onto nitrocellulose membrane for western blot analysis. Rabbit anti-myc antibodies were probed to detect ORF3 expression in transfected cells and immunoprecipitated sample. Mouse anti-β-actin and -myc antibodies were used to detect β-actin and myc IgG as internal controls.

**Figure 2:**
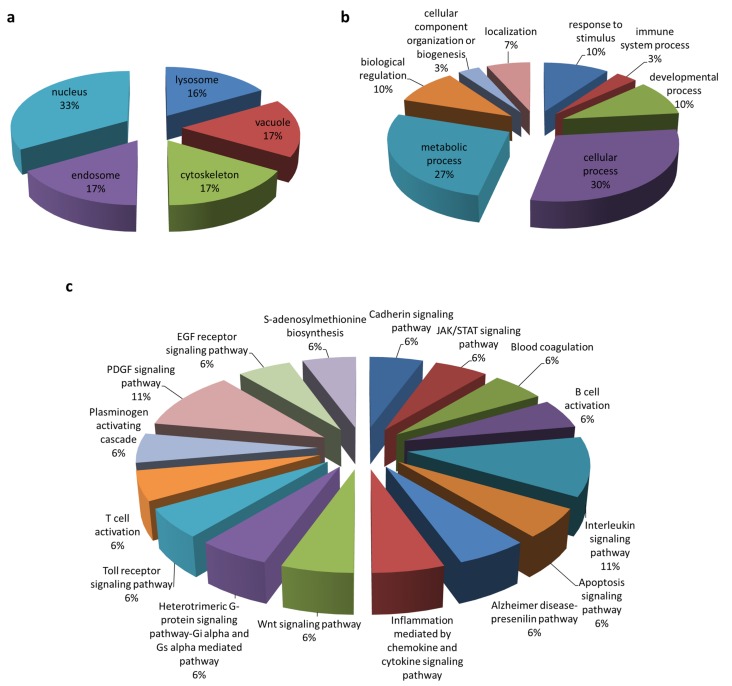
Gene annotation of the ORF3 interactome. Classification was based on (**a**) organelle compartment, (**b**) biological process, and (**c**) pathway by PANTHER analysis.

To screen for cellular protein partners of ORF3, immunoprecipitation coupled with mass spectrometry was performed using lysates of HEK293T cells overexpressing ORF3 with myc-tag at the C-terminus. The pull-down proteins were separated and analyzed by SDS-PAGE. Silver staining displayed several protein bands distinct from mock-transfected cells. Notably, the expression of ORF3 in cell lysates was confirmed by western blot (Figure 1). The LC-MS/MS data were analyzed to classify cellular protein partners using the NCBI nr database and the Mascot database search engines [17]. Gene ontology analysis was performed by PANTHER classification system [18,19]. The identified host proteins (Table 1) were classified based on organelle compartment and biological process (Figure 2). The majority of proteins pooled down with ORF3 were particularly localized in the endo-lysosomal organelles including endosome, lysosome, and vacuole (17%, 16%, and 17%, respectively) (Figure 2a). Gene ontology of identified proteins demonstrated that the proteins involved in various biological processes played a role in cellular endosomal trafficking, such as sorting nexin-13 (SNX13) and vacuolar protein-sorting-associated protein 36 (VPS36), and immune signaling pathways, such as signal transducer and activator of transcription 4 (STAT4) and inhibitor of nuclear factor kappa-B kinase subunit beta (IKBKB) (Figure 2b,c, Table S1). 

### 3.2. Validation of ORF3myc Interaction

**Figure 3:**
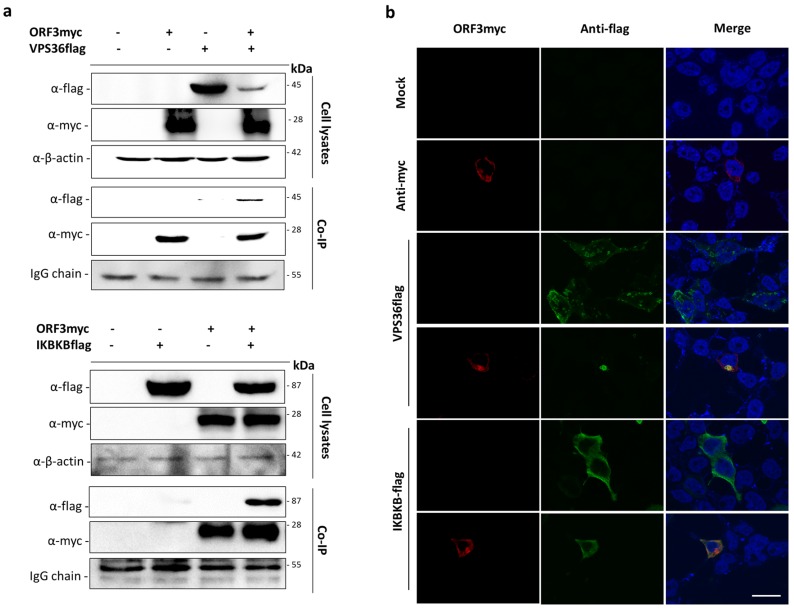
Validation of the interactions of ORF3 with VPS36 and IKBKB by co-immunoprecipitation and indirect immunofluorescence assay (IFA). (**a**) The plasmid expressing VPS36 or IKBKB with flag-tag at the C-terminus (pCAGGS_VPS36 or IKBKBflag) was co-transfected with pCAGGS_ORF3myc in HEK293T cells. At 24 hpt, cell lysates were subjected to immunoprecipitation using anti-myc bead. The immunoprecipitated proteins were probed with rabbit anti-flag and anti-myc antibodies. (**b**) The cells were incubated with mouse anti-myc and rabbit anti-flag as primary antibodies. Goat anti-rabbit IgG Alexa Fluor 488 and goat anti-mouse IgG Alexa Fluor 647 were used as secondary antibodies. Protein localization was analyzed by confocal microscopy. The scale bar is 10 μm.

Bioinformatic analysis of the interactome data suggested that ORF3 may be involved in different cellular processes, particularly in endosomal and immune signaling pathways. We therefore selected VPS36 and IKBKB as representative proteins to validate the interaction between ORF3myc and protein partners. The plasmid expressing VPS36 or IKBKB with flag-tag at the C-terminus was co-transfected with ORF3myc in HEK293T cells. Cell lysates were then subjected to co-immunoprecipitation. Likewise, transfected cells were analyzed for protein co-localization by immunofluorescence assay (IFA). The results showed that not only could ORF3 be pooled down with VPS36 and IKBKB, it also appeared co-localized with both proteins in the cytoplasm of transfected cells (Figure 3)

Proteins in the ESCRT-II complex are known to be essential for controlling the innate antiviral defense [20]. Moreover, previous studies in HIV and hepatitis B virus demonstrated that VPS36 plays multiple roles in regulating virus replication [21,22]. As the role of VPS36 in PEDV virus infection is lacking, we further investigated the role of VPS36 protein in the context of PEDV replication in subsequent experiments.

### 3.3. The GLUE Domain of VPS36 Is Not Required for Binding to Ubiquitinated ORF3

**Figure 4:**
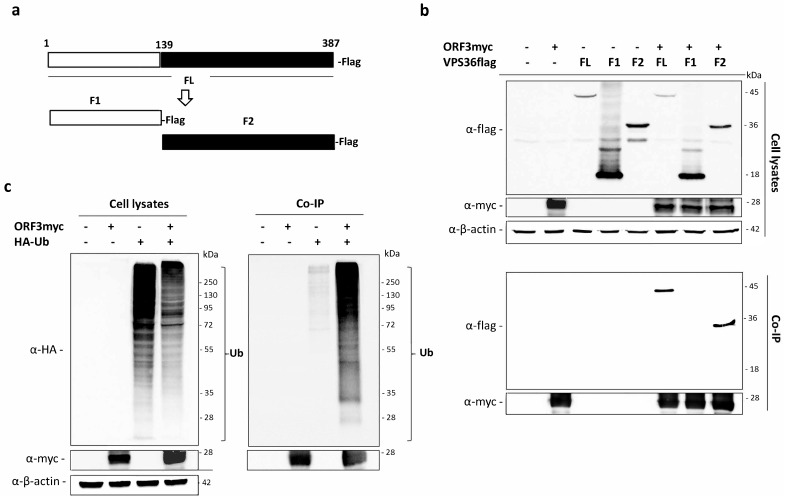
Ubiquitination of ORF3 and its binding with VPS36 domains. (**a**) Schematic representation of VPS36 domain constructs (full length; FL, fragment 1; F1 (amino acids 1–139) and fragment 2; F2 (amino acids 140–389). (**b**) The plasmid expressing ORF3myc and VPS36flag proteins were co-expressed in HEK293T cells. The protein complexes were immunoprecipitated using anti-myc bead. The immunoprecipitated proteins were probed with rabbit anti-flag and anti-myc antibodies. (**c**) The plasmid expressing ORF3myc and HA-Ub were co-transfected into HEK293T cells. Empty plasmid was used as a control. At 24 hpt, cleared lysates were subjected to immunoprecipitation using anti-myc bead. The immunoprecipitated proteins were probed with rabbit anti-HA and anti-myc antibodies.

The mammalian VPS36 is known to specifically bind to ubiquitin and sort the ubiquitinated proteins through the endo-lysosomal pathway for protein degradation [23,24]. The binding was typically through the “GRAM-like ubiquitin binding in EAP45” (GLUE) domain. To this end, we hypothesized that VPS36 might modulate ORF3 expression by interacting with ubiquitinated ORF3 through its GLUE domain. To examine whether ORF3 was ubiquitinated, we co-transfected HEK293T cells with the plasmid expressing ubiquitin with HA-tag at N-terminus (HA-Ub) and that expressing ORF3myc. The ORF3 was pulled-down by immunoprecipitation using anti-myc bead, and the ubiquitination was detected by western blotting against anti-HA tag. As depicted in Figure 4c, HA-ubiquitinated protein could be co-precipitated with ORF3, thereby suggesting that ORF3 was ubiquitinated in transfected HEK293T cells.

To further identify the region of VPS36 required for binding with the ORF3, we divided the full length VPS36 (FL) into 2 fragments (F1 and F2). F1 contained the GLUE domain (amino acids 1–139) as previously described [24] and F2 contained the VPS36 region devoid of the GLUE domain (amino acids 140–387) (Figure 4a). Co-immunoprecipitation was performed to examine physical interaction between ORF3 and VPS36 fragments. To our surprise, we detected that ORF3 did not bind to F1 but strongly bound to F2 (Figure 4b).

### 3.4. Overexpression of VPS36 Protein Suppresses PEDV Replication in VeroE6-APN Cells

**Figure 5:**
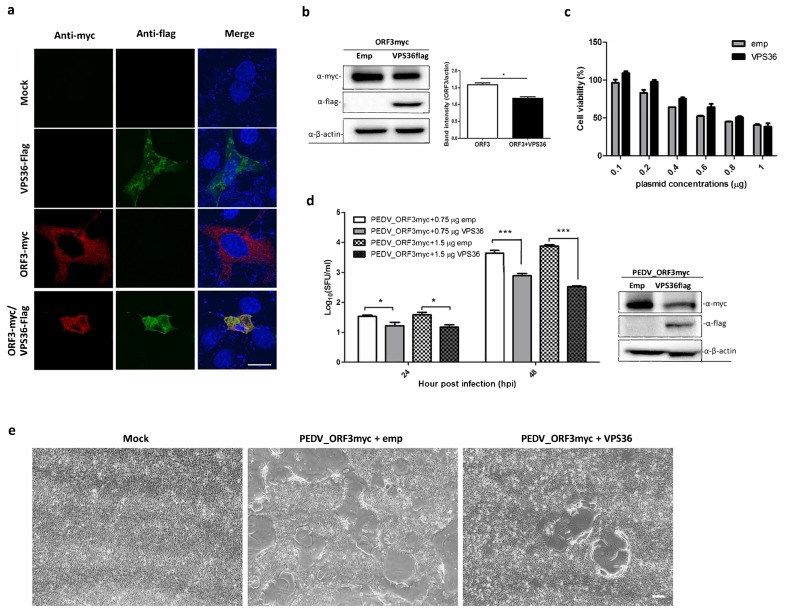
Co-expression of ORF3 and VPS36 and inhibitory effect of VPS36 to PEDVAV12_ORF3myc replication in VeroE6-APN cells. VeroE6-APN cells were transfected with the plasmids expressing ORF3 and VPS36 or mock transfected. At 24 hpt, cell lysates were subjected to (**a**) immunofluorescence staining to visualize protein co-localization and (**b**) western analysis to determine ORF3 expression level in transfected cells. (**c**) VeroE6-APN cells grown in a 96-well plate were transfected with plasmid expressing VPS36 at different concentrations. At 24 hpt, cell viability (CCK-8 assay) was performed. The absorbance data was compared between mock transfected and transfected cells. The results are expressed as the mean of three independent experiments ± standard error of means (SEM). (**d**) PEDV_AV12__ORF3myc growth kinetics in VPS36 overexpressed cells. The plasmid expressing VPS36 at different concentrations (0.75 and 1.5 µg) were transfected into VeroE6-APN cells. At 8 hpt, the cells were washed and infected with PEDV_AV12__ORF3myc at MOI of 0.1. Supernatants were collected for virus titration (SFU/mL) at indicated time points. *, *p* < 0.05 ***, *p* < 0.001. (**e**) Syncytia formation of PEDV_AV12__ORF3myc infected VeroE6-APN cells at 48 hpi. The scale bar is 200 µm.

Given that ORF3 directly interacts with VPS36, it is possible that VPS36 would destine ORF3 for degradation. To this end, we co-expressed ORF3 and VPS36 in VeroE6-APN cells and performed an IFA and a western blot analysis to examine protein co-localization and expression. As shown in Figure 5a,b, ORF3 was mainly co-localized with VPS36 in the cytoplasm and, notably, ORF3 expression was declined in the presence of VPS36. The reduction of ORF3 protein expression was unlikely due to cell cytotoxicity caused by VPS36 (Figure 5c). To further investigate whether VPS36-mediated reduction of ORF3 would affect PEDV replication, we determined the growth kinetics of PEDV_AV12__ORF3myc in VPS36-expressing VeroE6-APN cells. As expected, we found that VPS36 could suppress PEDV_AV12__ORF3myc virus replication in a dose-dependent manner (Figure 5d). Difference in cytopathic effect (CPE) was also apparent in the presence of VPS36 in infected cells (Figure 5e). Collectively, our data suggested that VPS36 could restrain PEDV_AV12__ORF3myc replication in VeroE6-APN cells.

### 3.5. Recovery of PEDV_AV12__ORF3myc Growth in VPS36 Knock-Down Cells

**Figure 6:**
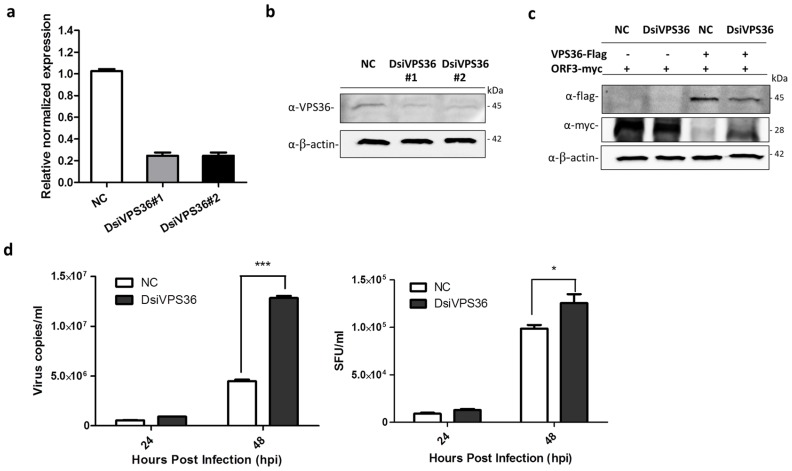
Silencing of VPS36 by DsiRNAs (DsiVPS36s). VeroE6-APN cells were transfected with each DsiVPS36 at a concentration of 40 nM. DsiRNA (NC) was used as negative control. At 48 hpt, two DsiRNAs targeting VPS36 were examined for their silencing activity by (**a**) RT-qPCR and (**b**) western blot analysis. For RT-qPCR, relative mRNA expression of VPS36 was normalized with the internal control GAPDH. Error bars indicate mean ± SEM from duplicate experiments. For western blot, rabbit anti-VPS36 antibodies were used to detect endogenous VPS36. (**c**) Plasmids expressing VPS36flag and ORF3myc were co-transfected into VeroE6-APN cells. At 8 hpt, cells were transfected with DsiVPS36_1 or NC and further incubated for 48 h. Rabbit anti-flag and -myc antibodies were used to detect exogenous VPS36 and ORF3. β-actin was used as internal control. (**d**) PEDV_AV12__ORF3myc growth kinetics in VPS36 knock-down cells. VeroE6-APN cells were infected with PEDV_AV12__ORF3myc at MOI of 0.1 after transfection with DsiVPS36 for 48 h. Error bars indicate mean ± SEM. Cell supernatants were collected for virus quantification (virus copies/mL) and viral titer (SFU/mL) at indicated time points. *, *p* < 0.05, *** denotes *p* < 0.001.

Small interfering RNA or silencing RNA (siRNA) is a common strategy used to study single gene function, which has been widely applied for exploring a role of viral-host protein interaction on virus life cycle in vitro and in vivo [25,26]. Our results so far are solely based on VPS36 overexpressing in host cells. To investigate whether the endogenously expressed VPS36 would have an inhibitory effect against PEDV_AV12__ORF3myc replication, we attempted to knock down the expression of VPS36 by DsiRNA targeting VPS36 (DsiVPS36). The DsiVPS36 sequences are shown in Table 2. VeroE6-APN cells transfected with each DsiVPS36 were subjected to RT-qPCR and western blot analysis to determine VPS36 mRNA and protein expression, respectively. The results shown in Figure 6a,b indicate that both DsiVPS36 could reduce VPS36 expression at mRNA and protein levels. Of note, DsiVPS36 was found to specifically target VPS36 but not the viral ORF3 (Figure 6c). Interestingly, when VeroE6-APN cells were transfected with DsiVPS36 and subsequently infected by PEDV_AV12__ORF3myc, the growth of PEDV_AV12__ORF3myc was higher in VPS36 knock-down cells when compared to the infected cells with intact VPS36 expression (Figure 6d). Notably, the difference in viral titer, albeit statistically significant, was less pronounced than those of viral copy number. Taken together, our findings suggest that endogenous VPS36 in VeroE6-APN cells also have suppressive effect against PEDV replication.

### 3.6. ORF3 Degradation Was Not Recovered in VPS36-Overexpressed Cells Treated with Lysosome Inhibitor

**Figure 7:**
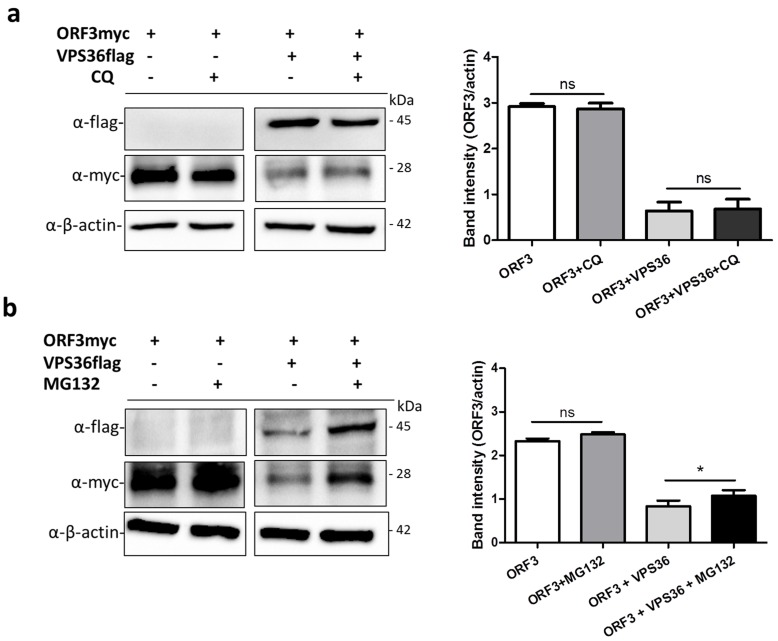
ORF3 expression in the presence of chloroquine and MG132. VeroE6-APN cells were transfected with the plasmids expressing ORF3 and VPS36 proteins. At 8 hpt, cell lysates were treated with chloroquine and MG132 at a concentration of 20 µM and 10 µM, respectively, for 1 h. At 48 hpt, cells were harvested and subjected to western blot analysis. Rabbit anti-flag and -myc antibodies were used to probe VPS36flag and ORF3myc proteins. *, *p* < 0.05.

VPS36, together with other ESCRTs components, functions by sorting membrane protein cargo into multivesicular bodies (MVBs) pathway, consequently leading to protein degradation by acidic hydrolases in the lysosome [27]. Chloroquine has been widely used to prevent endosomal and lysosomal acidification and restricted virus replication [28,29]. To this end, we tested whether ORF3 is targeted by VPS36 and destined for degradation in lysosome. The plasmids expressing ORF3myc and VPS36flag were co-transfected in VeroE6-APN cells treated with chloroquine. The result showed that chloroquine cannot relieve a negative effect of VPS36 on ORF3 downregulation (Figure 7a). However, using of MG132 to inhibit protein degradation through ubiquitin-proteasome pathway recovered the ORF3 expression in the VPS36-expressing cells (Figure 7b).

## 4. Discussion

Despite mounting experimental data underlying PEDV replication and pathogenesis, the function of the ORF3 accessory protein, especially its role in host-virus interaction, still needs more investigation. ORF3 has been shown to regulate PEDV replication in vitro and to play a key role in viral virulence in vivo [6,7,8,9]. Recently, we have shown that ORF3 is not only localized in the ER-Golgi compartments but also on the cell surface by interacting with the spike protein [9]. As the transport through the ER and Golgi apparatus is critical for protein biosynthesis and secretory pathway [30], the localization of ORF3 in these compartments points to the possibility that ORF3 might interact with various cellular proteins. In this study, we attempted to acquire further insights into ORF3 activity using a combined method of immunoprecipitation and mass spectrometry to discover cellular interacting proteins of ORF3. 

At least 22 proteins were identified as potential ORF3 interacting partners and selected candidates were further examined by co-immunoprecipitation to validate proteomics results. It should also be emphasized that proteins that weakly or transiently bind with ORF3 may not be identified in this study. Analyses by PANTHER database [18,31] revealed that the majority of ORF3-inteacting partners are those that are localized in the organelles of the endo-lysosomal pathway including endosome, lysosome, and vacuole. This finding is in line with our previous study showing ORF3 localization in ER-Golgi secretory compartments [9]. It is also important to note that analyses, based on the biological process and pathway, strongly suggest the role of ORF3 in immune signaling pathways such as JAK/STAT, T and B cell activation, and interleukin signaling pathways (Table S1). In particular, we identified the inhibitor of nuclear factor kappa-B kinase subunit beta (IKBKB) as an interacting partner of ORF3. This finding might at least in part explain the ability of ORF3 to inhibit NF-κB-mediated and IRF3-dependent type-I IFN production [32].

Most, if not all, coronaviruses utilized endo-lysosomal pathway for entering into host cells [26]. It is intriguing that several ORF3-interacting proteins such as SNX13, MCOLN2, and VPS36 are associated with this pathway. VPS36, in particular, has been reported to regulate viral entry and morphogenesis [21,22]. In this study, we showed that VPS36 could strongly interact with ORF3, leading to ORF3 downregulation, which, in turn, suppresses PEDV replication. As VPS36 is known to play a role for cargo sorting into MVBs to allow protein degradation when the MVBs fuse with lysosome [27], it could possibly explain our finding that ORF3 might be prone to VPS36-mediated degradation. Interestingly, although VPS36 usually binds to ubiquitin through the GLUE domain and sorts the ubiquitinated proteins for protein degradation [23,24], our results indicate that VPS36 mediates ORF3 degradation perhaps via a distinct mechanism. Specifically, we found that VPS36 lacking the GLUE domain, but not the fragment bearing the GLUE domain, could efficiently bind to ubiquitinated ORF3. These results thus point to other possibilities such as ubiquitin-independent ORF3-VPS36 interaction or interaction with other components in the ESCRT complex [33,34]. Another point worth mentioning is the fact that while lysosome inhibitor, chloroquine, failed to prevent the decrease of ORF3 expression, MG132, a proteasome inhibitor, could substantially alleviate ORF3 degradation, thereby suggesting that the ubiquitinated ORF3 might be targeted to the proteasome for degradation.

In the virus infection context, we observed that the downregulation of VPS36 by DsiRNAs could partially augment the viral yield. We are indeed disinclined to conclude that this effect is solely due to undisturbed ORF3 expression. It has been shown in our previous studies that ORF3 could suppress PEDV replication [7]. In addition, cell-adapted PEDV strains that replicate to high titer in vitro usually harbor abortive or truncated ORF3 [9]. In contrast, some studies showed conflicting results, showing that ORF3 could promote PEDV replication [6]. It is thus not clear whether the presence of intact ORF3 accounted for enhanced PEDV replication in our study. While VPS36–ORF3 interaction might play a role in regulating PEDV replication, we speculate that overexpression of VPS36 might affect PEDV replication via other mechanisms. Future studies will be needed to delineate the intracellular signaling pathway mediated by VPS36 in PEDV-infected cells and how VPS36 affects each step of PEDV replication.

In summary, this study successfully employed a proteomics-based approach to identify cellular partners of the ORF3 accessary protein. Among many candidates, VPS36 has been extensively investigated and identified as a potential suppressor of PEDV replication. A better understanding of how VPS36 interfere with PEDV replication is critical not only for our overall understanding of PEDV–host interaction, but for our general knowledge of how host proteins are modulated in the face of stress triggered by viral infection.

## Figures and Tables

**Table 1:** Identified proteins from immunoprecipitation reaction of ORF3 transfected cell lysates.

Protein Name; Gene Name	Binding Ratio
Human Glutathione Transferase O_2_; *GSTP1*	18.25905
ADAMTS-like protein 3; *ADAMTSL3*	18.0177
Sorting nexin-13; *SNX13*	17.84919
Zinc finger protein 443; *ZNF443*	17.36122
NDUFA13 protein; *NDUFA13*	17.18651
Frizzled-6; *FZD6*	17.17375
S-adenosylmethionine synthase isoform type-2; *MAT2A*	16.98951
Protein ripply3; *RIPPLY3*	16.88675
JAW1-related protein MRVI1B; *MRVI1B*	16.86521
Calmodulin isoform 2; *CALM1*	16.71276
Splicing factor, suppressor of white-apricot homolog; *SFSWAP*	16.53506
Signal transducer and activator of transcription 4; *STAT4*	16.40851
Pre-mRNA 3’-end-processing factor FIP1; *FIP1L1*	16.19049
Plasminogen; *PLAU*	15.85092
Inhibitor of nuclear factor kappa-B kinase subunit beta; *IKBKB*	15.5677
Mucolipin-2; *MCOLN2*	15.31623
protein TANC1 isoform X25; *TANC1*	15.10605
Vacuolar protein-sorting-associated protein 36; *VPS36*	14.34693
Probable E3 ubiquitin-protein ligase MARCH10; *MARCH10*	13.37567
Centrosomal protein of 44 kDa; *CEP44*	13.13018
ATP synthase mitochondrial F1 complex assembly factor 2 isoform X4; *ATPAF2*	12.90607
Xaa-Pro aminopeptidase 3; *XPNPEP3*	10.94451

**Table 2:** DsiRNA and primers used in this study.

DsiRNAs/Primers	Sequence
**DsiRNAs**	
DsiVPS36#1	5’-GUCAUGGUAAUUGAGCUUCAGUCUC-3’
	3’-CGCAGUACCAUUAACUCGAAGUCAGAG-5’
DsiVPS36#2	5’-AAGAUGAGACCAUCAGGUUUAAAUC-3’
	3’-UCUUCUACUCUGGUAGUCCAAAUUUAG-5’
Negative control DsiRNA (NC)	5’-CGUUAAUCGCGUAUAAUACGCGUAT-3’
	3’- CAGCAAUUAGCGCAUAUUAUGCGCAUA-5’
**Primers for RT-qPCR**	
VPS36_F	5’-GCCCTGGAGACAGTTTCAGA-3’
VPS36_R	5’-CTGAGTCATCACGGCAAAGA-3’
GAPDH_F	5’-CCCTTCATTGACCTCAACTACAT-3’
GAPDH_R	5’-ACGATACCAAAGTTGTCATGG-3’

## Supplementary Materials

The following are available online at https://www.mdpi.com/1999-4915/11/4/382/s1, Table S1: Pathway analysis of interacted host proteins by using PANTHER data base.

## References

[1] Debouck P., Pensaert M. (1980). Experimental infection of pigs with a new porcine enteric coronavirus, CV777. Am. J. Vet. Res..

[2] Sun R.Q., Cai R.J., Chen Y.Q., Liang P.S., Chen D.K., Song C.X. (2012). Outbreak of porcine epidemic diarrhea in suckling piglets, china. Emerg. Infect. Dis..

[3] Kocherhans R., Bridgen A., Ackermann M., Tobler K. (2001). Completion of the porcine epidemic diarrhoea coronavirus (PEDV) genome sequence. Virus Genes.

[4] De Haan C.A.M., Rottier P.J.M. (2005). Molecular interactions in the assembly of coronaviruses. Advances in virus research.

[5] Park S.J., Moon H.J., Luo Y., Kim H.K., Kim E.M., Yang J.S., Song D.S., Kang B.K., Lee C.S., Park B.K. (2008). Cloning and further sequence analysis of the ORF3 gene of wild- and attenuated-type porcine epidemic diarrhea viruses. Virus Genes.

[6] Wang K., Lu W., Chen J., Xie S., Shi H., Hsu H., Yu W., Xu K., Bian C., Fischer W.B. (2012). PEDV ORF3 encodes an ion channel protein and regulates virus production. FEBS Lett.

[7] Wongthida P., Liwnaree B., Wanasen N., Narkpuk J., Jongkaewwattana A. (2017). The role of ORF3 accessory protein in replication of cell-adapted porcine epidemic diarrhea virus (PEDV). Arch. Virol..

[8] Ye S., Li Z., Chen F., Li W., Guo X., Hu H., He Q. (2015). Porcine epidemic diarrhea virus ORF3 gene prolongs S-phase, facilitates formation of vesicles and promotes the proliferation of attenuated PEDV. Virus Genes.

[9] Kaewborisuth C., He Q., Jongkaewwattana A. (2018). The accessory protein ORF3 contributes to porcine epidemic diarrhea virus replication by direct binding to the spike protein. Viruses.

[10] Jitoboam K., Phaonakrop N., Libsittikul S., Thepparit C., Roytrakul S., Smith D.R. (2016). Actin interacts with dengue virus 2 and 4 envelope proteins. Plos One.

[11] Wu W., Tran K.C., Teng M.N., Heesom K.J., Matthews D.A., Barr J.N., Hiscox J.A. (2012). The interactome of the human respiratory syncytial virus NS1 protein highlights multiple effects on host cell biology. J. Virol..

[12] Kuo R.L., Chen C.J., Tam E.H., Huang C.G., Li L.H., Li Z.H., Su P.C., Liu H.P., Wu C.C. (2018). Interactome analysis of NS1 protein encoded by influenza a H7N9 virus reveals an inhibitory role of NS1 in host mRNA maturation. J. Proteome Res..

[13] Jengarn J., Wongthida P., Wanasen N., Frantz P.N., Wanitchang A., Jongkaewwattana A. (2015). Genetic manipulation of porcine epidemic diarrhoea virus recovered from a full-length infectious cDNA clone. J. Gen Virol..

[14] Taylor R.T., Best S.M. (2011). Assessing ubiquitination of viral proteins: Lessons from flavivirus NS5. Methods.

[15] Shevchenko A., Tomas H., Havlis J., Olsen J.V., Mann M. (2006). In-gel digestion for mass spectrometric characterization of proteins and proteomes. Nat. Protoc..

[16] Livak K.J., Schmittgen T.D. (2001). Analysis of relative gene expression data using real-time quantitative PCR and the 2−∆∆CT method. Methods.

[17] Perkins D.N., Pappin D.J., Creasy D.M., Cottrell J.S. (1999). Probability-based protein identification by searching sequence databases using mass spectrometry data. Electrophoresis.

[18] Mi H., Muruganujan A., Thomas P.D. (2013). Panther in 2013: Modeling the evolution of gene function, and other gene attributes, in the context of phylogenetic trees. Nucleic Acids Res..

[19] Mi H., Poudel S., Muruganujan A., Casagrande J.T., Thomas P.D. (2016). Panther version 10: Expanded protein families and functions, and analysis tools. Nucleic Acids Res..

[20] Kumthip K., Yang D., Li N.L., Zhang Y., Fan M., Sethuraman A., Li K. (2017). Pivotal role for the ESCRT-II complex subunit EAP30/SNF8 in IRF3-dependent innate antiviral defense. PLoS Pathog..

[21] Stieler J.T., Prange R. (2014). Involvement of ESCRT-II in hepatitis B virus morphogenesis. PLOS ONE.

[22] Meng B., Ip N.C., Prestwood L.J., Abbink T.E., Lever A.M. (2015). Evidence that the endosomal sorting complex required for transport-II (ESCRT-II) is required for efficient human immunodeficiency virus-1 (HIV-1) production. Retrovirology.

[23] Alam S.L., Langelier C., Whitby F.G., Koirala S., Robinson H., Hill C.P., Sundquist W.I. (2006). Structural basis for ubiquitin recognition by the human ESCRT-II Eap45 GLUE domain. Nat. Struct. Mol. Biol..

[24] Slagsvold T., Aasland R., Hirano S., Bache K.G., Raiborg C., Trambaiolo D., Wakatsuki S., Stenmark H. (2005). Eap45 in mammalian ESCRT-II binds ubiquitin via a phosphoinositide-interacting GLUE domain. J. Biol. Chem..

[25] Darniot M., Schildgen V., Schildgen O., Sproat B., Kleines M., Ditt V., Pitoiset C., Pothier P., Manoha C. (2012). RNA interference in vitro and in vivo using DsiRNA targeting the nucleocapsid N mRNA of human metapneumovirus. Antivir. Res..

[26] Burkard C., Verheije M.H., Wicht O., van Kasteren S.I., van Kuppeveld F.J., Haagmans B.L., Pelkmans L., Rottier P.J.M., Bosch B.J., de Haan C.A.M. (2014). Coronavirus cell entry occurs through the endo-/lysosomal pathway in a proteolysis-dependent manner. PLoS Pathog..

[27] Teo H., Perisic O., Gonzalez B., Williams R.L. (2004). ESCRT-II, an endosome-associated complex required for protein sorting: Crystal structure and interactions with ESCRT-III and membranes. Dev. Cell.

[28] Ooi E.E., Chew J.S., Loh J.P., Chua R.C. (2006). In vitro inhibition of human influenza A virus replication by chloroquine. Virol. J..

[29] Delvecchio R., Higa L.M., Pezzuto P., Valadão A.L., Garcez P.P., Monteiro F.L., Loiola E.C., Dias A.A., Silva F.J.M., Aliota M.T. (2016). Chloroquine, an endocytosis blocking agent, inhibits Zika virus infection in different cell models. Viruses.

[30] Gomez-Navarro N., Miller E. (2016). Protein sorting at the ER-Golgi interface. J. Cell Biol..

[31] Thomas P.D., Campbell M.J., Kejariwal A., Mi H., Karlak B., Daverman R., Diemer K., Muruganujan A., Narechania A. (2003). Panther: A library of protein families and subfamilies indexed by function. Genome Res..

[32] Zhang Q., Ma J., Yoo D. (2017). Inhibition of NF-kappaB activity by the porcine epidemic diarrhea virus nonstructural protein 1 for innate immune evasion. Virology.

[33] Frankel E.B., Audhya A. (2018). ESCRT-dependent cargo sorting at multivesicular endosomes. Semin. Cell Dev. Biol..

[34] Mageswaran S.K., Dixon M.G., Curtiss M., Keener J.P., Babst M. (2014). Binding to any ESCRT can mediate ubiquitin-independent cargo sorting. Traffic.

