# Supplementary material for: Porcine Epidemic Diarrhea Virus (PEDV) ORF3 Interactome Reveals Inhibition of Virus Replication by Cellular VPS36 Protein
Source: Viruses. 2019 Apr 24;11(4):382. doi: 10.3390/v11040382 (PMC6521123; doi:10.3390/v11040382)
Supplement: Supplementary file 1 [file viruses-11-00382-s001.pdf]

Supplementary tables

Table S1 Pathway analysis of interacted host proteins by using PANTHER data base

	Gene Name
**Biological processes**	
response to stimulus (GO:0050896)	IKBKB, STAT4, FZD6
immune system process (GO:0002376)	STAT4
developmental process (GO:0032502)	IKBKB, STAT4, RIPPLY3
cellular process (GO:0009987)	VPS36, FIP1L1, IKBKB, SFSWAP, SNX13, ADAMTSL3, MCOLN2, STAT4, RIPPLY3
metabolic process (GO:0008152)	VPS36, NDUFA13, FIP1L1, IKBKB, SFSWAP, ADAMTSL3, STAT4, RIPPLY3
biological regulation (GO:0065007)	IKBKB, FZD6, STAT4
cellular component organization or biogenesis (GO:0071840)	SFSWAP
localization (GO:0051179)	VPS36,SNX13
**Pathways**	
Cadherin signaling pathway (P00012)	FZD6
JAK/STAT signaling pathway (P00038)	STAT4
Blood coagulation (P00011)	PLG
B cell activation (P00010)	IKBKB
Interleukin signaling pathway (P00036)	IKBKB, STAT4
Apoptosis signaling pathway (P00006)	IKBKB
Alzheimer disease-presenilin pathway (P00004)	FZD6
Inflammation mediated by chemokine and cytokine signaling pathway (P00031)	IKBKB
Wnt signaling pathway (P00057)	FZD6
Heterotrimeric G-protein signaling pathway-Gi alpha and Gs alpha mediated pathway (P00026)	SNX13
Toll receptor signaling pathway (P00054)	IKBKB
T cell activation (P00053)	IKBKB
Plasminogen activating cascade (P00050)	PLG
PDGF signaling pathway (P00047)	IKBKB, STAT4
EGF receptor signaling pathway (P00018)	STAT4
S-adenosylmethionine biosynthesis (P02773)	MAT2A
